# Spatio-temporal dynamics of autophagy-associated genes in macrophage-driven atherosclerosis: an integrated omics and experimental study

**DOI:** 10.3389/fendo.2026.1764263

**Published:** 2026-02-26

**Authors:** Wenqi Cao, Binyang Wang, Yuxue Wang, Yunyan Li, Hanning Yang, Yue Sun, Lirong Xu, Yongping Lu

**Affiliations:** 1 Kunming Medical University, Kunming, Yunnan, China; 2 Department of Ultrasound, The Affiliated Hospital of Yunnan University, Kunming, Yunnan, China

**Keywords:** atherosclerosis, autophagy, immune microenvironment, macrophage, single-cell analysis

## Abstract

**Objective:**

Atherosclerosis (AS) is a leading cardiovascular disease driven by lipid metabolism dysregulation and immune maladaptation. Although macrophage autophagy modulates plaque stability, the specific autophagy-related genes governing AS progression, particularly in spatial and immune contexts, remain poorly defined. This study aimed to systematically identify and characterize key macrophage autophagy-associated genes in AS using an integrated multi-omics approach.

**Methods:**

Transcriptomic datasets (GSE270260, GSE100927) and single-cell RNA-seq data (GSE260657) were analyzed. Machine learning (LASSO and RF-SVM) screened for core autophagy-related genes. Their diagnostic value was evaluated using ROC and decision curve analysis. Immune infiltration, functional enrichment (GO/KEGG/GSEA), single-cell clustering, pseudotemporal trajectory analysis, and spatial transcriptomic mapping were performed. *In vitro* validation was conducted in ox-LDL-induced macrophages via qPCR, western blot, and immunofluorescence.

**Results:**

Three autophagy-related genes—SNX5, SMG1, and GSK3A—were identified as core regulators. They showed strong diagnostic potential for AS (combined AUC = 0.844) and correlated significantly with immune cell infiltration, particularly B cells and macrophages. Functional enrichment linked them to metabolic reprogramming and immune-inflammatory pathways, including NF-κB. Single-cell and spatial analysis revealed distinct expression patterns across plaque regions and cell types, with pseudotemporal trajectory indicating dynamic upregulation of GSK3A and SMG1 during macrophage-to-foam cell transition. *In vitro* experiments confirmed their upregulation at mRNA and protein levels upon ox-LDL induction.

**Conclusion:**

SNX5, SMG1, and GSK3A are pivotal regulators of macrophage lipid handling and immune modulation in AS, exhibiting dynamic spatiotemporal expression within plaques. These genes represent promising diagnostic biomarkers and potential therapeutic targets for stabilizing atherosclerotic plaques.

## 1 Introduction

Atherosclerosis (AS), a primary cause of coronary artery disease (CAD), represents the most prevalent form of cardiovascular disease (CVD). It is a leading contributor to myocardial infarction (MI) and stroke, accounting for significant morbidity and mortality worldwide (1). Studies indicate that the global prevalence of ischemic heart disease has increased by more than 80% over the past three decades. Economic development, accompanied by shifts in traditional dietary patterns, persistent tobacco use, and the growing burden of obesity, diabetes, and hypertension, has posed substantial public health challenges and elevated the risk of atherosclerotic cardiovascular events. MI and cerebral infarction resulting from AS continue to severely impact human health (2).Addressing the increasing incidence of AS among younger populations has become an urgent research priority.

The pathological essence of AS lies in a vicious cycle of lipid deposition and dysregulated immune responses (3). Macrophages are recognized as the predominant immune cell type within atherosclerotic lesions (4, 5).Their metabolic homeostasis is a critical determinant of plaque progression and stability. Atherosclerotic plaques consist of a necrotic core—comprising lipids, cellular debris, cholesterol crystals, macrophages, and foam cells—covered by a fibrous cap (6). Upon phagocytosis by macrophages, oxidized low-density lipoprotein (ox-LDL) activates Toll-like receptor 4 (TLR4), triggering the NF-κB signaling pathway. This promotes the release of pro-inflammatory factors such as IL-1β and TNF-α, and recruits additional monocytes to the site. Apoptosis of foam cells activates the NLRP3 inflammasome, leading to IL-18 and IL-1β maturation, and enhances infiltration by neutrophils and mast cells. The continual recruitment of immune cells amplifies local inflammation and undermines the structural integrity of the fibrous cap. Sustained inflammation further suppresses collagen synthesis and facilitates metalloproteinase-9 (MMP-9)-mediated degradation of the cap, ultimately predisposing the plaque to rupture (7, 8). Therefore, promoting lipid clearance and modulating inflammatory responses represent key therapeutic strategies for restoring macrophage metabolic balance and stabilizing atherosclerotic plaques. Macrophage autophagy plays an important role in metabolism and inflammation regulation.

Autophagy encompasses three primary forms: macroautophagy, microautophagy, and chaperone-mediated autophagy (9).Macroautophagy (hereafter referred to as autophagy) is an intracellular degradation process that facilitates the turnover of proteins, organelles, and cytoplasmic components in response to nutrient deprivation or cellular stress (10). It enables cells to adapt to fluctuating nutritional and energetic demands by recycling cellular material to maintain metabolic homeostasis and promote survival. Additionally, autophagy modulates inflammatory responses through interactions with innate immune signaling pathways, elimination of endogenous inflammasome agonists, and regulation of immune mediator secretion (11). Growing evidence indicates that autophagic flux plays a significant role in the pathogenesis of various diseases, including cardiovascular disorders, diabetes, inflammatory conditions, infections, and cancer. Furthermore, autophagy is closely related to AS.

Autophagy within macrophages has been increasingly implicated in the pathogenesis of vascular diseases (12). In the early stages of AS, macrophage autophagy exerts a protective effect by modulating key cellular processes, including the suppression of inflammation and apoptosis, and the promotion of cholesterol efflux. In the advanced stage of the disease, autophagic flux is often impaired or dysregulated. This functional deficiency compromises the clearance of damaged organelles and protein aggregates (such as p62), which in turn exacerbates oxidative stress, activates the NLRP3 inflammasome, and promotes the secretion of pro−inflammatory cytokines, thereby accelerating plaque necrosis and destabilization (13, 14). Supporting this biphasic role, studies in ApoE–/– mice show autophagic markers (p62 and LC3) colocalizing with plaque macrophages (15). Critically, p62 accumulation—a hallmark of reduced autophagic flux often accompanied by increased LC3-II—not only reflects this impairment but also actively exacerbates inflammation by activating the NF-κB pathway, thereby fueling a vicious cycle of plaque progression (16) (17). Given that hundreds of genes regulate autophagy, how their spatiotemporal expression dynamics precisely govern macrophage fate throughout these stages remains largely unexplored.

This study aims to systematically clarify for the first time the spatiotemporal dynamic expression and function of macrophage autophagy-related genes in AS by integrating multi-omics analysis and experimental verification. We focus on screening and verifying the key autophagy genes (SNX5, SMG1, GSK3A) that regulate plaque progression, and analyzing their core roles in lipid transport of macrophages, regulation of the immune microenvironment, and transformation of foam cells, with the aim of providing new perspectives for disease mechanism research and potential novel biomarkers and intervention strategies for early diagnosis and targeted therapy. This study has been reviewed by the Ethics Committee of Yunnan University (No.20230427).

## 2 Materials and methods

### 2.1 Data acquisition and processing

As-related datasets were retrieved from the Gene Expression Omnibus (GEO) database (https://www.ncbi.nlm.nih.gov/geo/), including bulk-tissue mRNA sequence data [GSE270260 (18), GSE100927 (19)] and scRNA-seq data [GSE260657 (20)]. A total of 15 human with carotid stenosis were included (n = 7, asymptomatic; n=8, symptomatic). 580 autophagy-related genes were obtained from the KEGG Pathway database.

### 2.2 Identification of DEGs and autophagy-related genes

The dataset GSE270260 was analyzed using the R package “DESeq2” (P < 0.05 and |log2(FoldChange)| >1). Differentially expressed genes (DEGs) were identified using volcano plots. Venn diagrams were used to identify overlapping genes between DEGs and autophagy gene sets. Two machine learning algorithms, Lasso and RF-SVM, were used to further screen effective overlapping genes, and the target difference genes were obtained by intersection of the two.

### 2.3 Key gene validation and prediction efficacy evaluation

The dataset GSE270260 was analyzed using the R package “DESeq2” (21). The expressions of key genes were extracted from the dataset GSE100927, and the Wilcoxon test was used to calculate and visualize the expression difference between AS and control samples. We extracted the significant expressed genes. Based on the results of LASSO-Cox regression analysis, the R package “rms” (v 6.3-0) was used to build the nomogram models to predict the incidence of atherosclerotic plaques. The receiver operating characteristic (ROC) curve and decision curve analysis (DCA) were used to evaluate the discrimination ability and prediction effect of nomograms.

### 2.4 Immune infiltration analysis

Based on a gene set of 28 immune-related cells, the immune activity of each sample was evaluated by the ssGSEA algorithm of the R package”GSVA” (22).The disparity of immune infiltration between the two groups and their correlations were analyzed. We also use boxplot to visualize the abundance of immune cells in patients with plaque. Furthermore, the association between the level of immune infiltration and the expression of autophagy-related hub gene was investigated.

### 2.5 Enrichment analysis of biological pathways and functions

Kyoto Encyclopedia of Genes and Genomes(KEGG) and Gene Ontology (GO) enrichment analyses for biological processes and pathways were conducted using the R package clusterProfiler. Additionally, Gene Set Enrichment Analysis (GSEA) was performed with GSEA software (v4.1.0). We use GSEA to analysis the single autophagy-related genes’ enrichment analysis and pick the top 10 item to visualize.

### 2.6 scRNA−seq analysis

For single-cell characteristics investigations, we analyzed the scRNA-seq dataset GSE260657, following the “Seurat”standard procedure (23). This dataset has undergone mitochondrial gene removal and doublet elimination. We extracted genes expressed in over 3 cells or with cumulative read counts exceeding 300. The top 2,000 highly variable genes were used for principal component analysis (PCA) of variation. The Harmony algorithm was applied to remove batch effects across samples. Uniform Manifold Approximation and Projection (UMAP) was employed for dimensionality reduction visualization with 15 dimensions. After iterative subpopulation visualization at different resolutions, a resolution of 0.01 was selected, yielding six distinct subclusters. Cluster identities were annotated through AddModuleScore visualization using aggregated gene expression patterns. Simultaneously, autophagy activity was scored per cluster using the AddModuleScore function with an autophagy gene set. Boxplots were generated to visualize expression patterns of autophagy-related genes across different cell clusters. For trajectory analysis, the extracted subpopulations were processed using Monocle2 (24), involving re-embedding, batch effect correction, and re-clustering. UMAP visualization was performed on the processed data, followed by cluster annotation via the AddModuleScore method.

To reconstruct pseudotemporal ordering of cells, the Monocle 2 package in R was utilized. This approach facilitates the characterization of transcriptional dynamics among distinct cell types and clusters. The resultant cell trajectories, represented as a branched tree structure, were visualized in two-dimensional space following dimensionality reduction using the DDRTree method. Genes displaying significant expression changes across pseudotime within clusters were identified by applying the differentialGeneTestfunction.

### 2.7 Expression of the key genes in macrophages

Foam cell formation was then induced in RAW264.7 cells by treating them with 50 μg/ml ox-LDL in serum-free RPMI 1640 medium for 18 hours. The mRNA and protein expression levels of the key genes were analyzed using quantitative PCR (qPCR), Western blotting, and immunofluorescence staining.

#### 2.7.1 Quantitative PCR

Total RNA was isolated from cells with TRIzol reagent. Complementary DNA (cDNA) was synthesized using the 2× Universal Blue SYBR Green qPCR Master Mix (Servicebio, G3326-05). Quantitative real-time PCR was carried out on a 9600 PCR system with TB Green^®^ Premix Ex Taq™ II (TaKaRa, RR820A). The thermal cycling protocol consisted of an initial step at 95°C for 30 s, followed by 40 cycles of denaturation at 95°C for 5 s and annealing/extension at 60°C for 30 s. Gene expression levels were normalized to GAPDH as the reference gene, and relative quantification was performed using the 2−ΔΔCT method. All primer sequences used are provided in **Table 1**.

**Table 1:** Primer sequences.

Primer	Sequence
SMG1 F	CATGGCAGTCGTGCATTAGC
SMG1 R	TCATCTTCCCGGGTGATCCT
SNX5 F	TTCTTTCCTCGCACAGCCAC
SNX5 R	CGATCTGAAGCGATGGGTCA
GSK3A F	AAGCGTCAGTCGGGGCTAT
GSK3A R	ACTTCTTGGGAACGCTCTGG
M-GAPDH F	TGTGTCCGTCGTGGATCTGA
M-GAPDH R	GAGTTGCTGTTGAAGTCGCA

#### 2.7.2 Western blotting

Macrophages were collected and lysed using RIPA Lysis and Extraction Buffer supplemented with protease inhibitor, followed by centrifugation. The resulting supernatants were subjected to immunoblotting as previously described. Briefly, 25 μg of protein per sample was separated by 10% SDS-PAGE and transferred onto a PVDF membrane. Non-specific binding sites were blocked with 5% bovine serum albumin in Tris-buffered saline containing 0.1% Tween 20. The membranes were then incubated overnight with the following primary antibodies: SMG1 (Bioss, bsm-62379R), SNX5 (Huamei Bio, CSB-PA045108), GSK3A (Huamei Bio, CSB-PA05369A0Rb), and monoclonal mouse anti-β-actin (Proteintech, Cat# 60004-1-1g). In addition, the protein expression levels of LC3 (Affinity, AF7001) and P62 (BIOSS, bs-8878R) were assessed to evaluate autophagic flux, with β-actin (Proteintech, 66009-1-Ig) serving as the loading control for normalization. Protein band images were quantified using ImageJ software (v1.52, NIH, USA).

### 2.8 Statistical analysis

All statistical analyses were conducted with R software (version 4.1.2). Group comparisons of continuous variables were carried out using the Wilcoxon test, while associations between variables were assessed via Pearson correlation analysis. Data visualization was performed with the “ggplot2” package. A two-sided P-value of less than 0.05 was considered statistically significant. Significance levels are indicated as follows: NS, not significant; *P < 0.05, **P < 0.01, and ***P < 0.001.

## 3 Results

### 3.1 Identification of DEGs and autophagy-related genes

**Figure 1:**
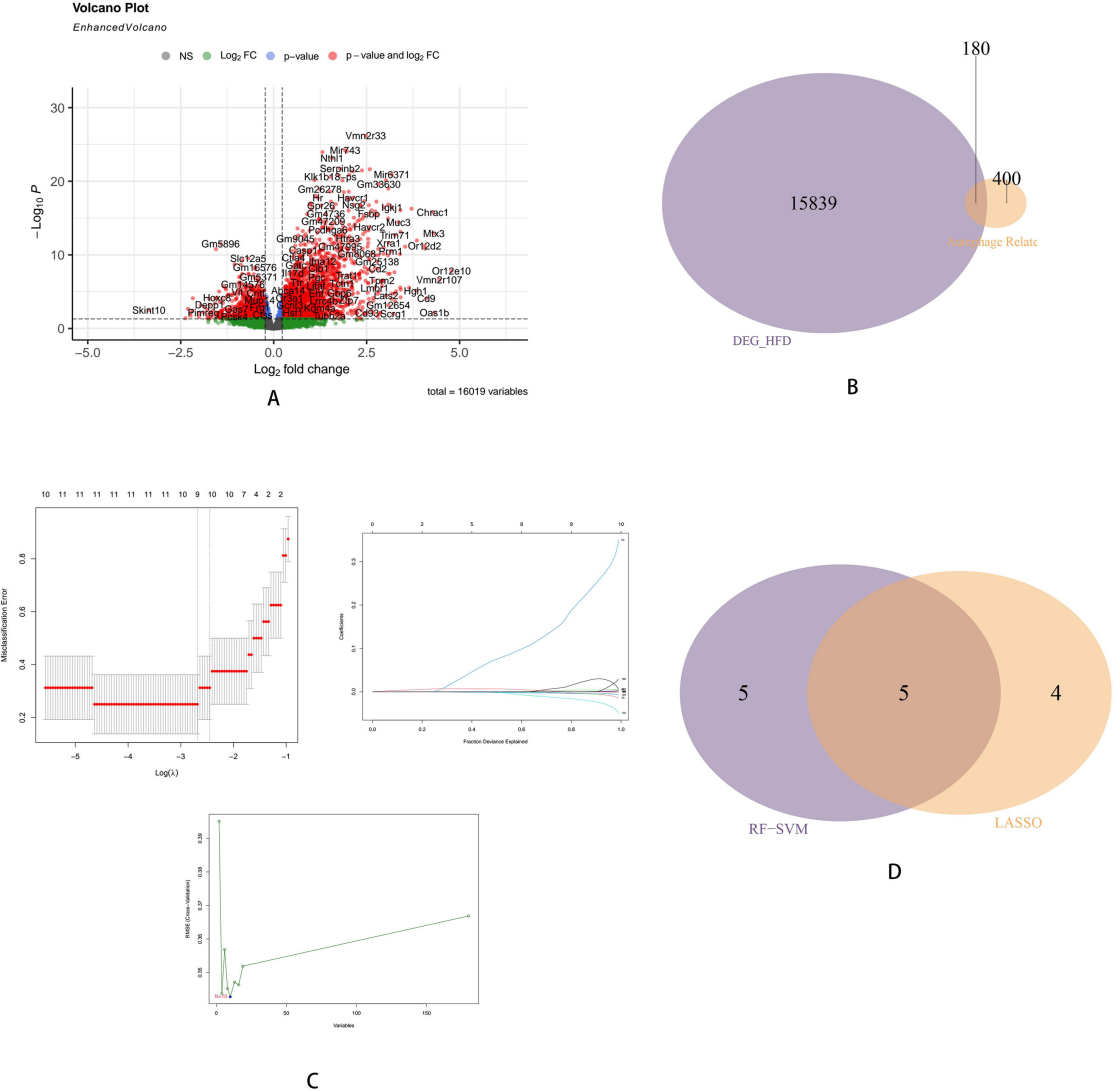
Identification of DEGs and autophagy-related genes. **(A)** The volcano plot displays the expression patterns of DEGs in AS. **(B)** The Venn diagram displays the shared genes between autophagy gene set and DEGs. **(C)** Lasso regression model and RF-SVM model to identify candidate genes in AS. **(D)** The Venn diagram shows the shared genes screened by LASSO regression model and RF-SVM model.

Based on the expression profile of GSE270260, differential expression analysis revealed that there were 16019 DEGs between HFD group and control group, of which 1534 genes were up-regulated and 2170 genes were down-regulated. We use ‘EnhancedVolcano’ to draw volcano plot to visualize the DEGs (**Figure 1A**). Vene diagram reveals 180 overlapping genes between DEGs and autophagy gene set (**Figure 1B**). In order to filter the value of predictive capability of overlapped genes, We use machine learning algorithm to do. LASSO regression and RF-SVM algorithm were further applied to identify key differential genes (**Figure 1C**). LASSO regression model identified 9 key differential genes, RF-SVM algorithm identified 10 key differential genes, and 5 overlapping genes (SNX5, SMG1, RNF41, KDM4A, GSK3A) were found after intersection (**Figure 1D**). The expression levels of these five genes associated with autophagy may change during AS.

### 3.2 Key gene validation and prediction efficacy evaluation

**Figure 2:**
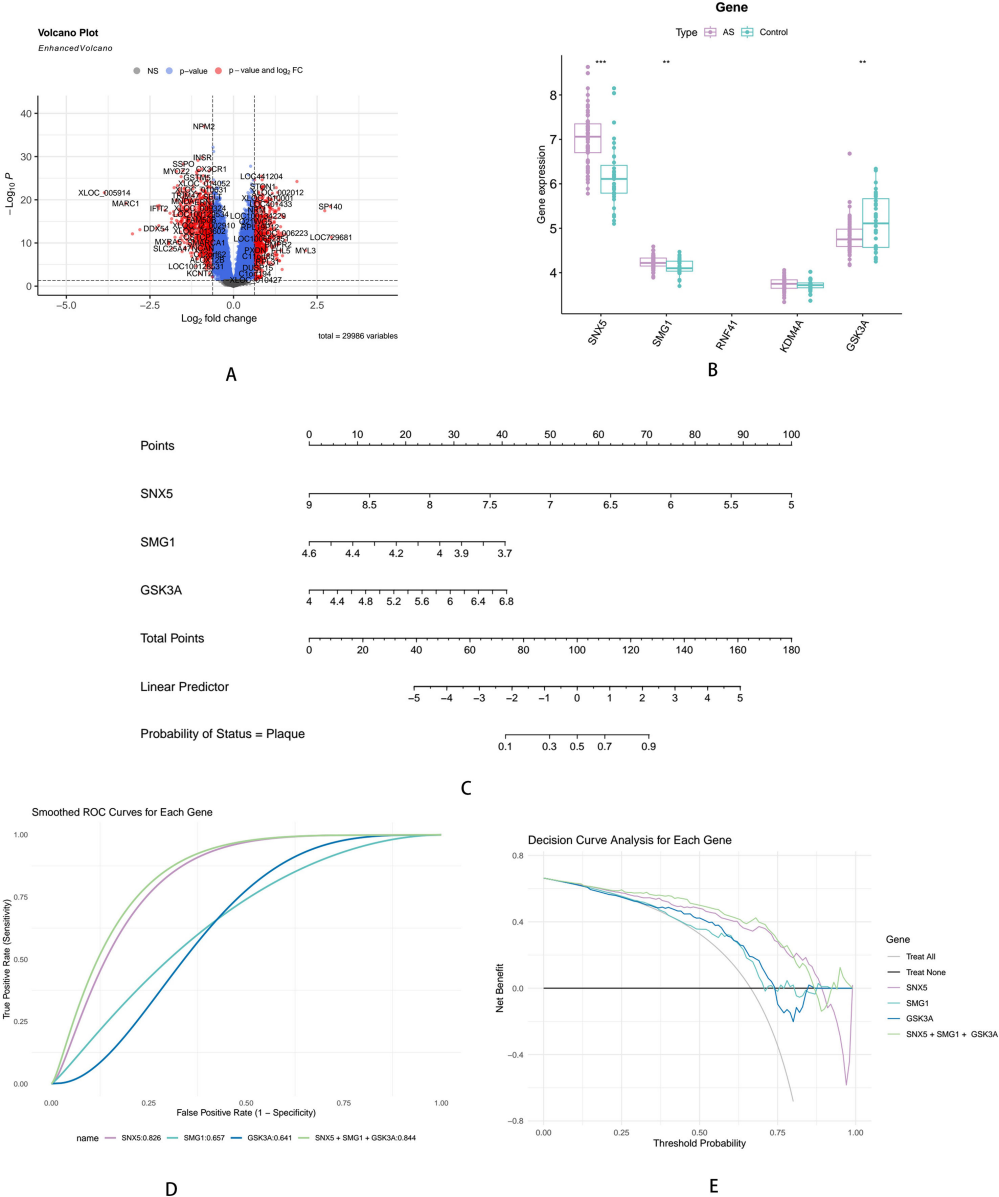
Key gene validation and prediction efficacy evaluation. **(A)** The volcano plot of DEGs. **(B)** The box plot of key genes expression differences. **(C)** The nomogram of key genes in AS diagnosis. **(D)** The ROC of key genes in AS diagnosis. **(E)** The DCA of key genes in AS diagnosis. **P<0.01, ***P<0.001.

External validation was performed using the independent dataset GSE100927, and volcano plots revealed a total of 29986 genes that differed between atherosclerotic and normal tissues, of which 200 were significantly up-regulated and 300 significantly down-regulated (|log2FC|>1, adj.*P* < 0.05). We use ‘EnhancedVolcano’ to draw volcano plot to visualize the DEGs (**Figure 2A**). The expression of 5 differential genes (SNX5, SMG1, RNF41, KDM4A, GSK3A) was compared between groups. The expression of SNX5 and SMG1 was significantly up-regulated and the expression of GSK3A was significantly down-regulated in AS group (**Figure 2B**). We construct the nomogram by those three autophagy-related genes showed that the lower the SNX5, SMG1 correspondence value, the higher the GSK3A correspondence value, the greater the probability of atherosclerotic plaque formation, and the change of SNX5 had a greater impact on the total number of points and the final probability (**Figure 2C**). We also draw three genes’ ROC curve respectively. The result showed that SNX5 has the best predictive capability (AUC = 0.826), the AUC of SMG1 is 0.657, and the GSK3A is the lowest AUC (0.641). But the combination of three genes’ predictive capability (AUC = 0.844) is superior than single gene’s predictive (**Figure 2D**). DCA showed that when the threshold probability ranged from 0.1 to 0.8, both the three biomarkers alone and in combination predicted plaque occurrence. In addition, the combination of SNX5, SMG1 and GSK3A was superior to SNX5, SMG1 and GSK3A alone in predicting plaque occurrence (**Figure 2E**).

### 3.3 Immune-related autophagy in AS

**Figure 3:**
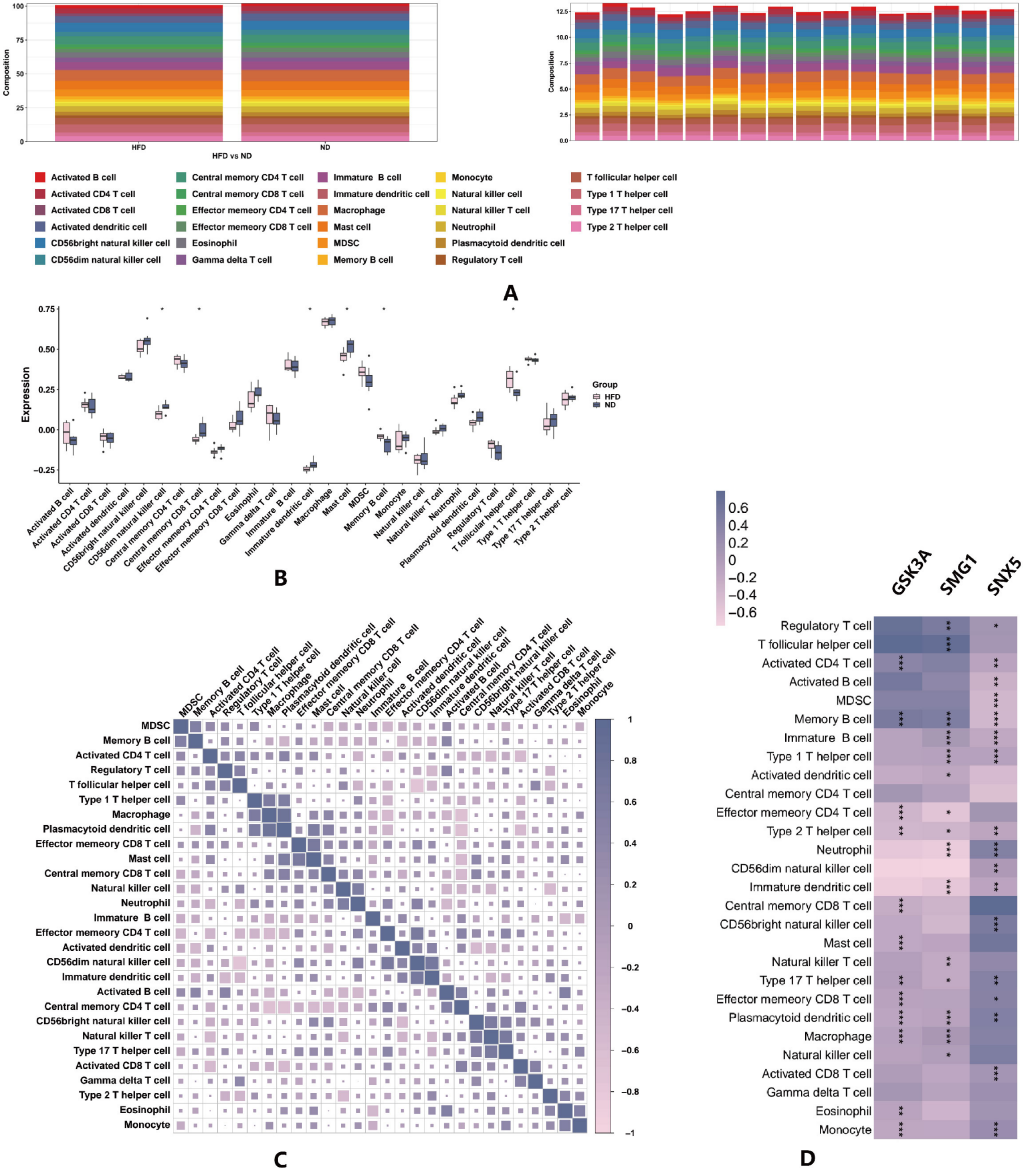
DEGs and immune cell correlation analysis. **(A)** The stacked bar plot of immune cell proportions. **(B)** The box line plot of immune cell proportion differences. **(C)** The heat map of immune cell interaction. **(D)** The heatmap of correlations between key genes and differential immune cells. *represents *P* < 0.05, **represents *P* < 0.01, ***represents *P* < 0.001, purple represents a positive correlation, and pink represents a negative correlation.

To elucidate on the subtypes of various immune cells, a quantitative analysis of the relative abundance of 28 types of immune cells in the 16 samples were achieved using the ssGSEA algorithm. Stacked area plot showed that the abundance of different immune cells. We use stacked bar plot to visualize the comparison of the abundance, it shows that CD56 natural killer cell, central memory CD8 T cell, immature dendritic cell, mast cell memory B cell, regulatory T cell and T follicular helper cell are significant (**Figures 3A, B**). Heat map reveals the interactions of immune cells during AS (**Figure 3C**). The darker the color and the larger the square, the stronger the correlation between the two cells. We evaluated the correlation between key differential genes (SNX5, SMG1 and GSK3A) expression and immune cell infiltration (**Figure 3D**).These autophagy-related differential genes were moderately correlated with immune cells such as Immune B cell, Memory B cell, macrophage and plasnacrytoid dendritic cell. Autophagy in immune cells is important for cellular immunity, differentiation, and survival.

### 3.4 GSEA enrichment analysis

SNX5, SMG1, and GSK3A are involved in diverse biological processes including immune regulation, cellular development, and metabolism. GSEA of KEGG pathways and GO terms further elucidated their functional associations.

**Figure 4:**
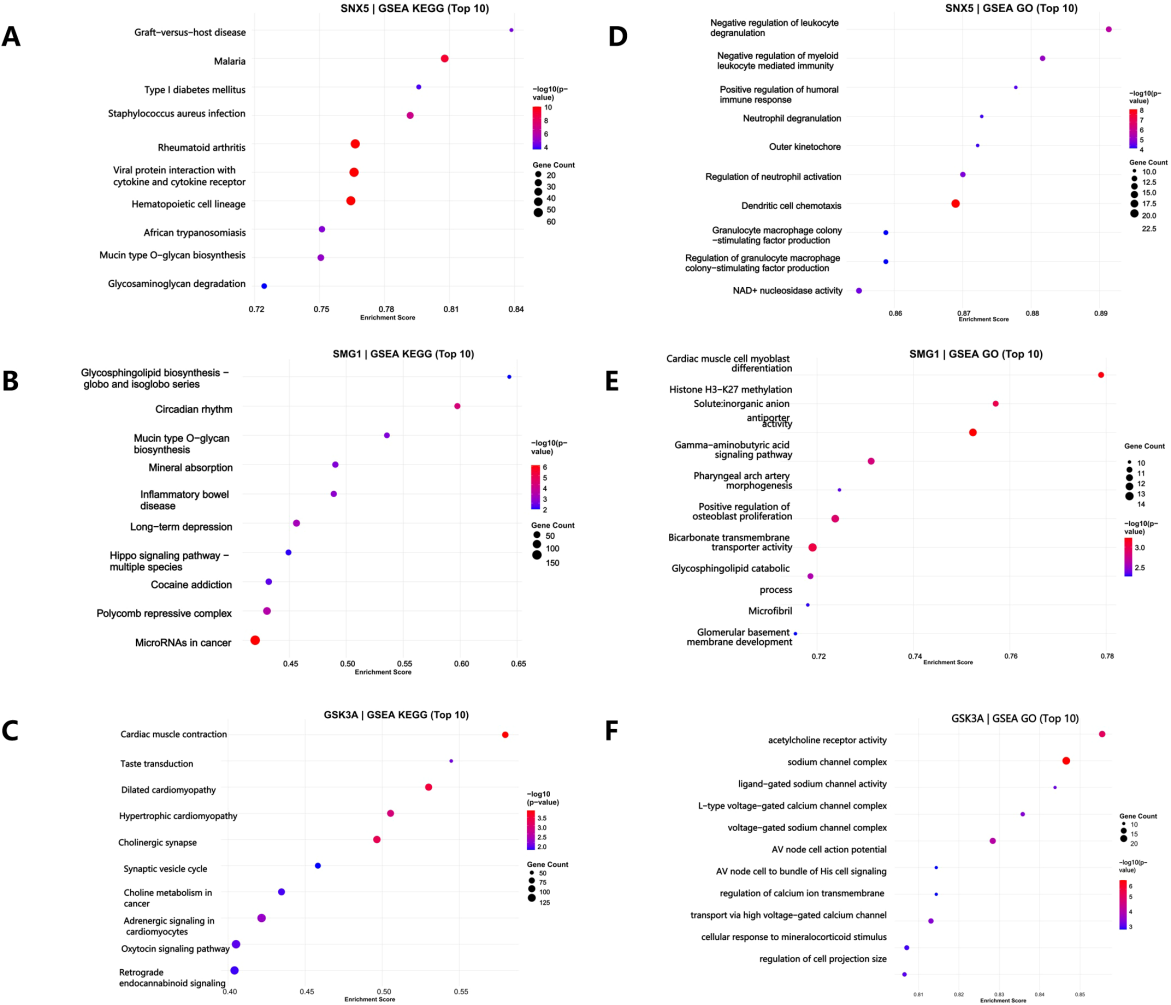
Gene Set Enrichment Analysis (GSEA) of key genes. **(A–C)** Bubble charts of the top 10 enriched KEGG pathways for SNX5 **(A)**, SMG1 **(B)**, and GSK3A **(C)**. **(D–F)** Bubble charts of the top 10 enriched GO biological processes for SNX5 **(D)**, SMG1 **(E)**, and GSK3A **(F)**. Bubble size represents the number of genes in each enriched pathway (gene count), and color intensity indicates the statistical significance. The x-axis shows the enrichment score.

SNX5 was enriched in KEGG pathways related to glycosaminoglycan degradation, mucin-type O-glycan biosynthesis, African trypanosomiasis, and rheumatoid arthritis (**Figure 4A**). GO analysis indicated involvement in NAD+ nucleosidase activity, regulation of granulocyte−macrophage colony-stimulating factor production, dendritic cell chemotaxis, and neutrophil degranulation (**Figure 4D**).

SMG1 showed KEGG enrichment in microRNAs in cancer, polycomb repressive complex, cocaine addiction, Hippo signaling, and mucin-type O-glycan biosynthesis (**Figure 4B**). Relevant GO terms included glomerular basement membrane development, glycosphingolipid catabolism, positive regulation of osteoblast proliferation, and cardiac muscle cell differentiation (**Figure 4E**).

GSK3A was associated with KEGG pathways such as retrograde endocannabinoid signaling, adrenergic signaling in cardiomyocytes, cholinergic synapse, and hypertrophic cardiomyopathy (**Figure 4C**). GO analysis suggested roles in calcium ion transmembrane transport, voltage−gated sodium channel complex, and acetylcholine receptor activity (**Figure 4F**).

### 3.5 Single−cell level expression of autophagy

To investigate autophagy at single-cell resolution within human atherosclerotic plaques, we conducted single-cell RNA sequencing (scRNA-seq) analysis. After standard quality control and filtering, 7,690 high-quality cells were retained. Dimensionality reduction and clustering resolved six distinct cell populations: Smooth Muscle Cells/Myofibroblasts, Macrophages, Endothelial Cells, and T Cells/B Cells. Cell type annotation was performed using established marker genes: B cells (CD19, CD79A, MS4A1, CD22, CD24, IGKC), Endothelial cells (PECAM1, VWF, CD34, CLDN5, FLT1, PLVAP), Macrophages (CD68, CD163, FCGR3A, FCGR1A, ITGAM, ITGAX, MRC1), Myofibroblasts (ACTA2, COL1A1, COL3A1, FN1, POSTN, FAP, THY1), Smooth muscle cells (ACTA2, MYH11, TAGLN, CNN1, DES, TPM2), and T cells (CD3D, CD3E, CD3G, CD4, CD8A, CD88, IL7R).

**Figure 5:**
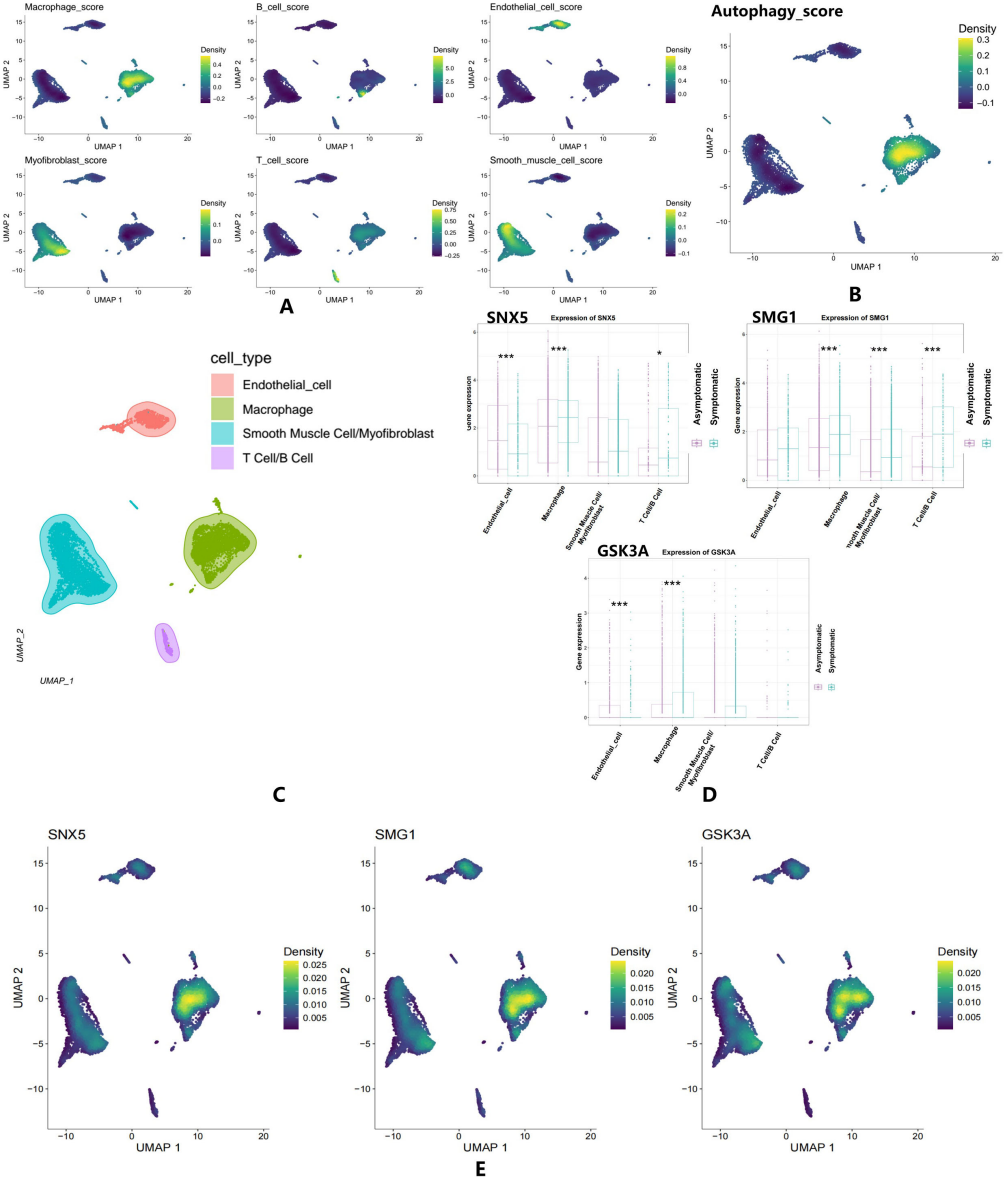
Single-cell level expression of autophagy. **(A)** The Density plot of Autophagy Gene Set Scores in six distinct cell populations. **(B)** The Density plot of Autophagy Gene Set Scores in AS cell population. **(C)** The cluster diagram of six cell populations. **(D)** The box plot for key genes expression in four cell types. **(E)** The density plot showed prominent co-expression of all three autophagy genes in macrophages. The lighter the green, the higher the density. *P < 0.05, ***P < 0.001.

Marker gene set scores were computed using Seurat’s AddModuleScore function. Density plots visualizing these scores revealed higher pathway activity in corresponding cell types, indicated by deeper green coloration (**Figures 5A, B**). Subsequent deconvolution analysis resolved expression patterns of three autophagy-related genes (SNX5, SMG1, GSK3A) across cell populations: SNX5 exhibited significant downregulation in endothelial cells but upregulation in macrophages; SMG1was upregulated in macrophages, smooth muscle cells/myofibroblasts, and T cells; GSK3A showed downregulation in endothelial cells with concurrent upregulation in macrophages (**Figures 5C, D**). Density plot analysis confirmed prominent co-expression of all three autophagy genes in macrophages, prompting their isolation for subsequent subcluster characterization (**Figure 5E**).

### 3.6 Pseudotime and trajectory analysis in macrophage

**Figure 6:**
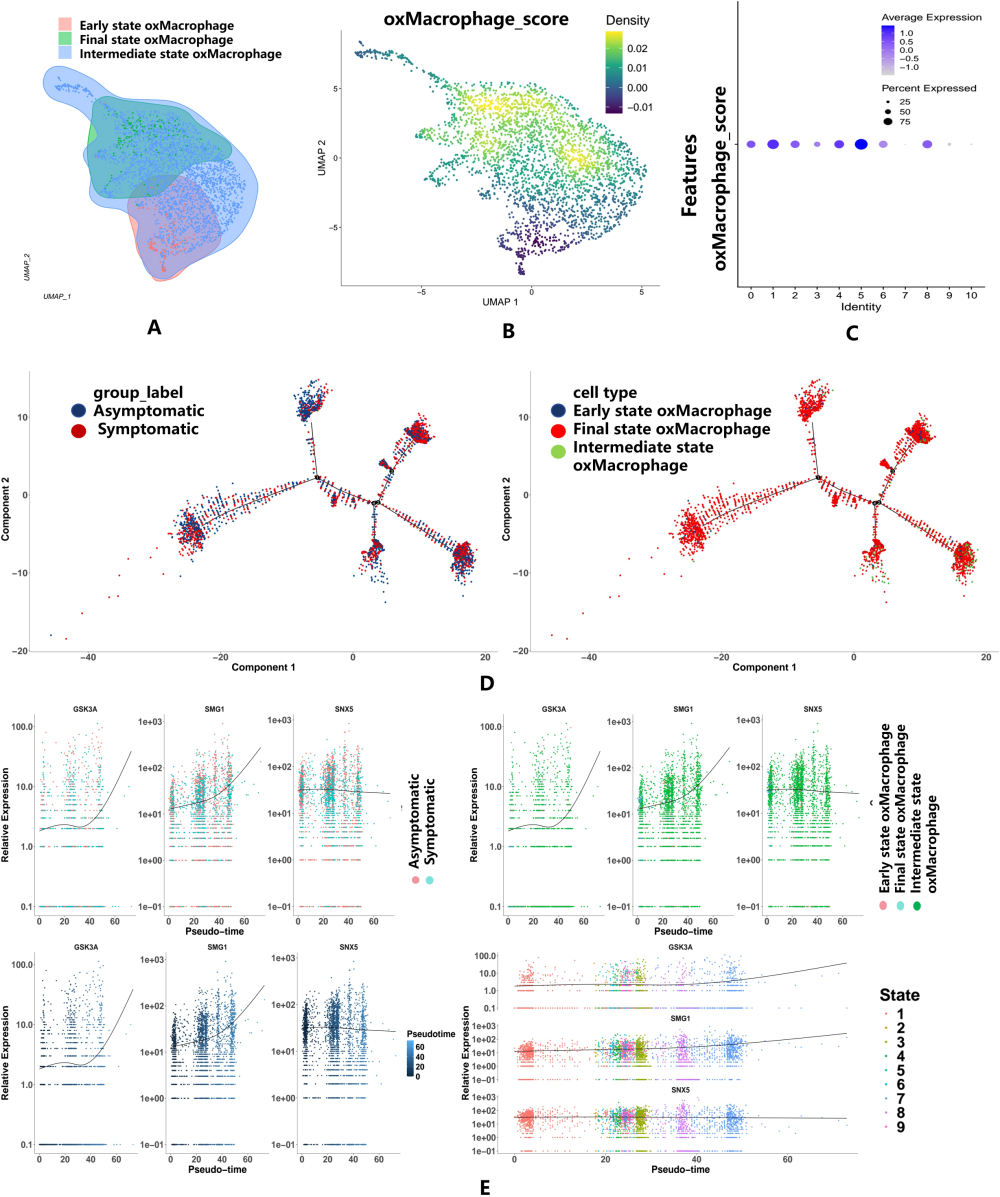
Pseudotime and Trajectory Analysis in macrophage. **(A)** The cluster diagram shows that the macrophage population divided into three different progressive foam clusters. **(B)** The density plot shows the distribution of foam cells in the macrophage population. **(C)** The dotpot display distinct maturation states of foam macrophages. **(D)** Macromimetic time series analysis. **(E)** The scatter plot of key gene expression in different states.

To delineate foam cell transition dynamics within the macrophage population, we performed subclustering analysis (**Figure 6A**). Macrophage subsets were stratified by dimensionality reduction and reclustering, with foam cell propensity quantified via Seurat’s AddModuleScore function using established markers (APOE, TREM2, CD36). The UMAP visualization revealed a continuous spectrum of scores, suggesting distinct maturation states of foam macrophages within the heterogeneous population (**Figures 6B, C**). Dot plot analysis confirmed three discrete subsets: Early-stage oxidized macrophage (Early oxMac), Intermediate-stage oxidized macrophage (Intermediate oxMac), and Late-stage oxidized macrophage (Late oxMac). Pseudotemporal ordering with Monocle2 resolved a differentiation trajectory across these states. When mapped against clinical symptom status, the trajectory demonstrated progressive enrichment of Late oxMac in symptomatic cases (**Figure 6D**). Analysis of autophagy gene dynamics along this trajectory revealed significant pseudotime-dependent upregulation of GSK3A and SMG1, while SNX5maintained stable expression with marginal decline (**Figure 6E**). Critically, the monotonic increase of GSK3Aand SMG1expression during macrophage-to-foam cell transition demonstrates their dynamic upregulation throughout foam cell maturation.

### 3.7 Spatial single transcriptomic analysis

**Figure 7:**
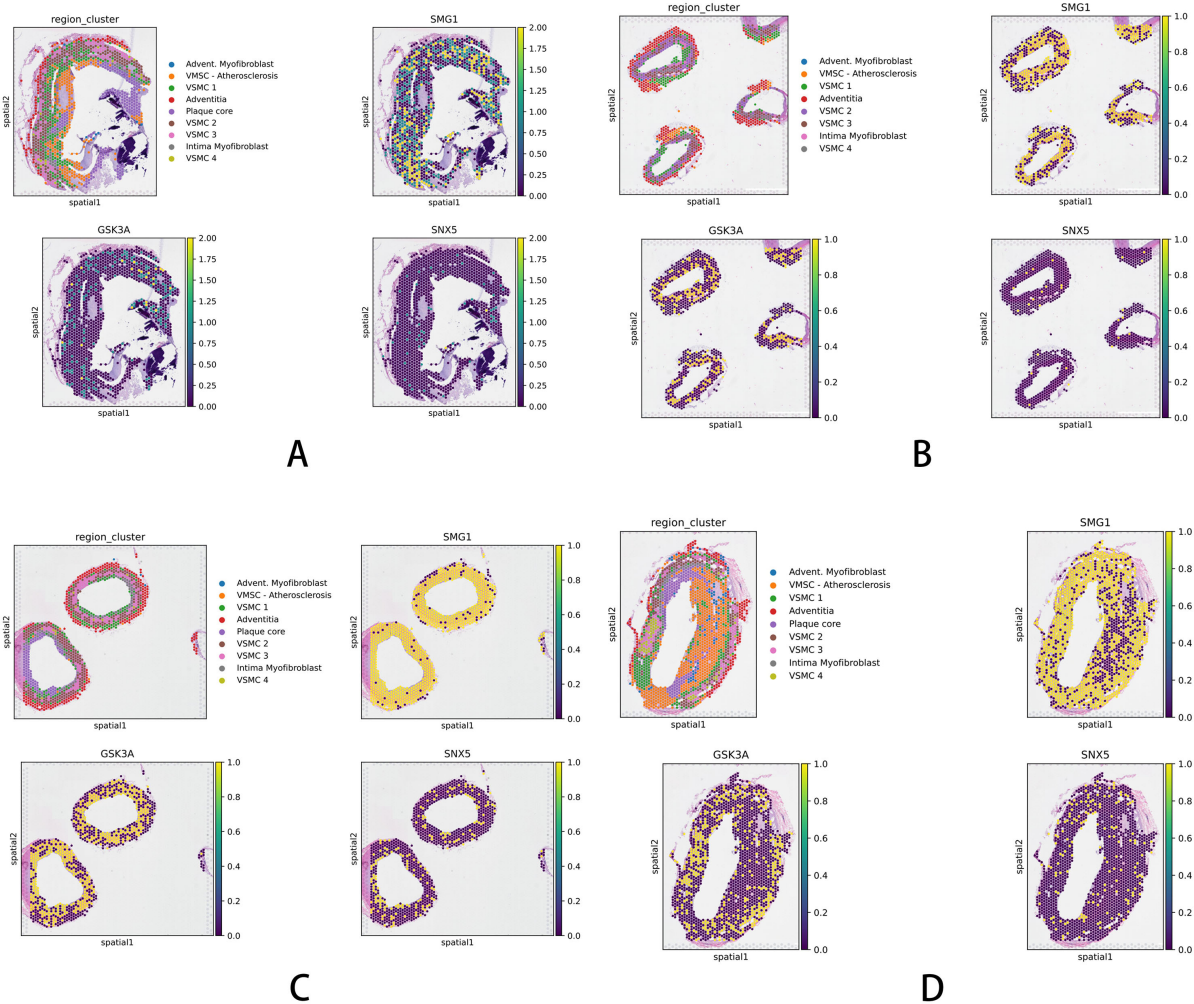
Spatial transcriptomic profiling of three key autophagy-related genes in an atherosclerotic plaque. **(A)** SMG1 was broadly expressed in both the plaque core and peripheral regions, as well as in vascular smooth muscle cells (VSMCs),GSK3A was predominantly localized to the core region, SNX5 showed minimal expression in the core. **(B)** SMG1 expression remained pronounced in the periphery and VSMCs,SNX5 expression remained low. **(C)** GSK3A exhibited a spatial expression pattern similar to SMG1, with detectable expression in the periphery and VSMCs, though at lower levels. **(D)** SNX5 and GSK3A have the consistent expression pattern, They are enriched in VSMC-rich areas.

Spatial transcriptomic analysis delineated distinct expression patterns for each gene. SMG1 expression was broad, encompassing the plaque periphery, core, and VSMCs. GSK3A was primarily enriched in the core, and SNX5 was largely absent from this region (**Figures 7A, B**). Quantitatively, SMG1 levels were highest in the periphery and VSMCs. Although sharing a similar spatial distribution, GSK3A expression was comparatively lower. SNX5 demonstrated overall low expression but was relatively enriched in VSMCs (**Figures 7C, D**).

### 3.8 Verification of key gene expression *in vitro*

**Figure 8:**
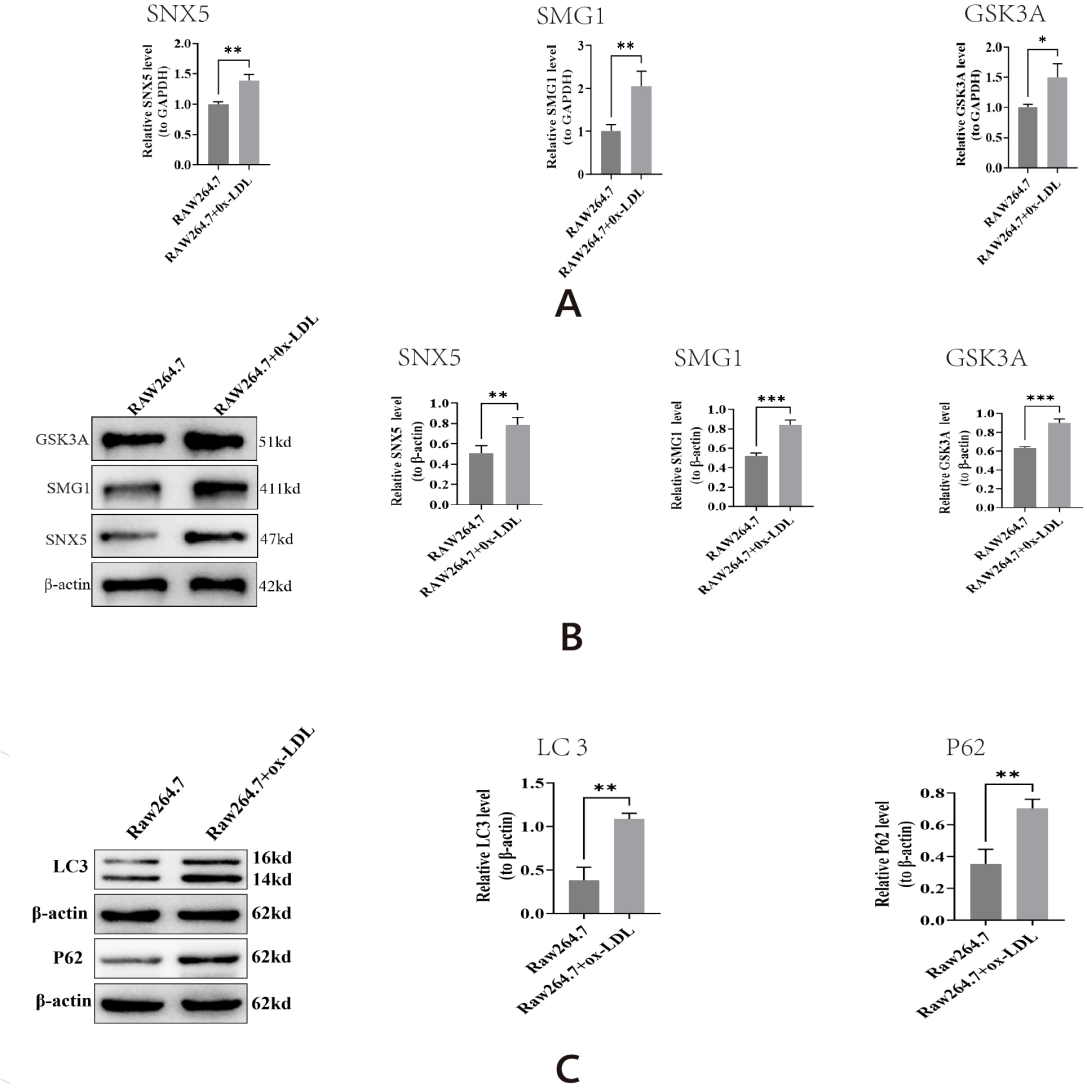
Expression detection of key genes in AS macrophages. **(A)** statistical graph of SNX5, SMG1, GSK3A mRNA expression level. **(B)** The SNX5, SMG1, GSK3A protein expression levels: Left, typical western blots, Right, statistical graph. **(C)** Protein expression of autophagy markers LC3 and p62. *P < 0.05, **P < 0.01, ***P < 0.001 vs. Raw264.7.

Using qPCR and Western blot analysis, we found that in an ox-LDL-induced *in vitro* model of AS, the mRNA and protein levels of SNX5, SMG1, and GSK3A were significantly upregulated compared to the control group (*P* < 0.05) (**Figures 8A, B**). Concurrently, this model exhibited dysfunctional autophagic flux, characterized by the accumulation of LC3-II and p62 proteins (**Figure 8C**). These results indicate that the upregulation of SNX5, SMG1, and GSK3A is closely associated with ox-LDL-induced autophagy dysregulation.

## 4 Discussion

AS is a chronic inflammatory disease driven by disordered lipid metabolism, primarily involving endothelial cells, vascular smooth muscle cells, and macrophages. Autophagy serves as an essential mechanism for degrading cytoplasmic components and maintaining cellular homeostasis (25).Macroautophagy enables cells to adapt to changes in nutrient and energy conditions by recycling intracellular components, thereby supporting metabolic homeostasis and cell survival (26). While the role of autophagy in AS has been widely studied in vascular smooth muscle cells and endothelial cells, its functions in macrophages—the key immune cells in atherosclerotic lesions—are still not fully understood. A key event in AS pathogenesis is the accumulation of ox-LDL, which has been shown to induce autophagy in macrophages (27). Evidence suggests that autophagy plays a stage-dependent role in AS. During the intermediate phase, macrophage autophagy exerts protective effects by inhibiting foam cell formation. Ox-LDL promotes autophagy via endoplasmic reticulum stress, facilitating the clearance of damaged organelles and proteins and enhancing macrophage survival. Moreover, autophagy contributes to cholesterol efflux through lysosomal degradation of lipid droplets, thereby reducing lipid accumulation. It also promotes polarization toward the M2 anti-inflammatory macrophage phenotype, supporting plaque stability (28). In contrast, as AS advances, autophagic activity becomes progressively impaired. This dysfunction leads to defective mitochondrial clearance, exacerbated lipid accumulation, increased macrophage death, and expansion of the necrotic core, ultimately accelerating plaque destabilization (29). Against this backdrop, the present study aimed to delineate the spatial expression patterns of autophagy-related genes in atherosclerotic tissues and to decipher their immunomodulatory roles using an integrated multi-omics approach. Our findings provide mechanistic insights into how autophagy regulates macrophage metabolic homeostasis, offering new perspectives for diagnostic biomarker development and immune-targeted therapies for AS.

Our comprehensive analysis identified SNX5, SMG1, and GSK3A as three autophagy-related genes whose expression is dynamically regulated during progression and spatially organized within human atherosclerotic plaques. This study, for the first time, integrates multi-omics approaches with experimental validation to demonstrate that SNX5, SMG1, and GSK3A form a core gene module that dynamically regulates macrophage fate in AS. These genes are not only upregulated in response to ox-LDL stimulation at both the transcriptional and protein levels, but they are also significantly associated with immune infiltration patterns and lipid metabolism reprogramming within plaques. Single-cell and spatial transcriptome analyses further elucidate the specific expression and spatiotemporal trajectory of this module across various macrophage subsets and plaque regions, providing essential molecular insights into the stage-dependent role of macrophage autophagy in AS. These findings offer new perspectives for enhancing plaque stability through the targeting of autophagy pathways.

SNX5, a member of the sorting nexin family, is a key regulator involved in endosomal sorting and intracellular trafficking. Beyond its role as a critical “transport supervisor” governing endocytosis and receptor degradation, aberrant expression of SNX5 has been implicated in the development, invasion, and metastasis of multiple human cancers. In macrophages, SNX5 serves as an essential regulator of macropinocytosis; its knockdown results in a 60–70% reduction in soluble antigen uptake efficiency (30). Consistently, Tian et al. demonstrated that SNX5 depletion in HeLa cells markedly inhibited the internalization of tetrahedral DNA nanostructures (31). Previous studies also associate SNX5 with clinical outcomes and signaling modulation across cancer types. For instance, SNX5 predicts poor prognosis and facilitates hepatocellular carcinoma progression via the EGFR-ERK1/2 pathway (32).In clear cell renal cell carcinoma(ccRCC), SNX5 is downregulated and correlates negatively with tumor size, clinical stage, venous tumor thrombus, and overall survival. Functionally, SNX5 inhibits TGF-β–induced epithelial–mesenchymal transition by interfering with CD44 endocytosis and trafficking, thereby suppressing ccRCC proliferation and metastasis (33). In thyroid cancer, SNX5 expression decreases with loss of differentiation—high in well-differentiated tumors and lost in poorly differentiated ones—and is linked to unfavorable prognosis. SNX5 deficiency disrupts trafficking of the TSH receptor and EGFR, leading to aberrant retention and sustained activation of downstream cAMP/PKA, MAPK, and AKT pathways, which drive tumor proliferation and malignant progression (34). Targeting SNX5 may provide new strategies for cancer treatment and metastasis prevention. In the context of AS, our study revealed elevated SNX5 expression and its correlation with immune responses. However, its precise role within the AS immune microenvironment remains unclear. Intriguingly, recent evidence indicates that SNX5 is essential for virus-induced autophagy, mitigating viral transmission and host damage via mechanisms seemingly independent of its canonical endocytic functions. Furthermore, SNX5 can suppress IL-10 production (11).We hypothesize that SNX5 may promote lipid endocytosis, suppress protective autophagy, and inhibit IL-10, collectively exacerbating inflammatory responses and contributing to atherosclerotic plaque formation.

SMG1, a key member of the phosphoinositide 3-kinase-related kinase family, functions as a tumor suppressor in various human malignancies (35). Notably, SMG1 has been shown to inhibit proliferation, migration, and invasion in ovarian cancer cells (36). Beyond its role in oncology, SMG1 regulates cholesterol synthesis and efflux in cancer cells through modulation of P53 isoforms (P53β/γ), underscoring its influence over metabolic homeostasis (35). This functional versatility positions SMG1 as a critical node linking tumor progression and metabolic reprogramming, highlighting its potential as a therapeutic target. Accumulating evidence also implicates SMG1 in the maintenance of genomic integrity and inflammatory regulation. SMG1 depletion compromises DNA damage repair capacity, resulting in the accumulation of oxidative damage (37). SMG1 loss contributes to a pro-inflammatory microenvironment by upregulating NF-κB-dependent cytokines, including IL-1β, IL-6, and IL-13 (38). Similarly, SMG1 negatively modulates inflammatory responses in innate immune cells by inhibiting Regnase-1 degradation (39). SMG1 was found to be highly expressed in AS, possibly as a result of cellular stress and compensatory responses. Ox-LDL stimulated mitochondrial dysfunction, ROS burst, a large number of pro-inflammatory factors released. SMG1 undergoes compensatory upregulation to combat hyperinflammation, maintain homeostasis, and prevent excessive cell death or DNA damage.

GSK3 is a serine/threonine protein kinase that regulates diverse cellular processes including glycogen metabolism, signal transduction, cell cycle progression, and proliferation (40). It exists in two isoforms, GSK3A and GSK3B. GSK3 is implicated in oxidative stress regulation across multiple diseases such as Alzheimer’s disease, cancer, diabetes, inflammatory disorders, and cardiovascular conditions (41–43). GSK3A is associated with various immune cell functions, and KEGG enrichment analysis indicates its strong involvement in inflammatory pathways including NF-κB. Zheng et al. identified GSK3A as a key facilitator of immune escape in liver cancer, promoting tumor growth in immunocompetent mice. Targeting GSK3A reverses immunosuppression and enhances PD-1 inhibitor efficacy (44). Additionally, GSK3A is essential for male fertility by maintaining sperm motility through regulation of PP1γ2 activity and energy metabolism (45). Consistent with previous studies, we found that GSK3A is overexpressed in a cellular model of AS, associated with increased oxidative stress, ongoing inflammatory response, and immune response modulation.GSK3 inhibitors have been investigated as potential treatments for a variety of diseases, including Alzheimer’s, bipolar disorder, diabetes, inflammatory diseases and some cancers (46).

Our multi-omics data suggest that SNX5, SMG1, and GSK3A—though functionally distinct in membrane trafficking, stress response, and inflammation—constitute an integrated “sensing–response–execution” network in atherosclerotic plaques. This network orchestrates macrophage responses to lipid overload: SMG1 senses ox-LDL stress and potentially modulates GSK3A to amplify NF-κB signaling, while SNX5 reprograms endocytic pathways to enhance lipid uptake. Furthermore, this cooperative axis likely underlies the observed autophagic impairment. SNX5 may perturb autophagosome formation or fusion, whereas SMG1- and GSK3A-driven signaling may inhibit autophagic degradation, resulting in characteristic p62 and LC3-II accumulation. Together, these interactions not only promote inflammation and lipid accumulation but also disrupt autophagy, thereby accelerating macrophage dysfunction and plaque progression.

In summary, we propose a hypothetical model: In the context of AS, SNX5, SMG1 and GSK3A do not operate in isolation but jointly shape the pro-atherosclerotic phenotype of macrophages by regulating the axis of lipid transport - metabolic stress - inflammatory signaling. This study provides new insights, yet there are notable limitations. The analysis primarily identified a robust correlation between SNX5, SMG1, and GSK3A expressions and the atherosclerotic phenotype, but the exact causal mechanism remains unclear. Experimental validation relies mainly on cell models, which cannot fully replicate the intricate multicellular *in vivo* microenvironment and long-term disease progression. Constraints in resolution and depth of techniques like spatial transcriptomics and single-cell sequencing, along with inadequate analysis at the protein modification level, may impact the precise interpretation of findings. The clinical translational potential of these genes as biomarkers or therapeutic targets necessitates further confirmation through prospective cohort studies and pharmacological intervention trials.

## 5 Conclusion

This study, for the first time, adopted an integrated multi-omics strategy to systematically reveal the key roles of autophagy-related genes SNX5, SMG1 and GSK3A in AS. These three genes constitute a molecular module that functions synergistically in lipid metabolism, stress response and inflammatory signaling in macrophages. Their expression has distinct spatiotemporal dynamic characteristics and is closely related to the remodeling of the plaque immune microenvironment. Our work not only provides a new molecular map for understanding the mechanism of macrophage dysfunction in the progression of AS, but also identifies a set of potential multi-target intervention hubs and diagnostic marker combinations, laying an important theoretical foundation for the future development of more precise AS diagnosis and treatment strategies.

## Data Availability

The datasets presented in this study can be found in online repositories. The names of the repository/repositories and accession number(s) can be found in the article/supplementary material.
